# Artificial Intelligence and Digital Technologies Against Health Misinformation: A Scoping Review of Public Health Responses

**DOI:** 10.3390/healthcare13202623

**Published:** 2025-10-18

**Authors:** Angelo Cianciulli, Emanuela Santoro, Roberta Manente, Antonietta Pacifico, Savino Quagliarella, Nicole Bruno, Valentina Schettino, Giovanni Boccia

**Affiliations:** 1 Department of Medicine, Surgery and Dentistry ‘’Scuola Medica Salernitana”, University of Salerno, 84081 Salerno, Italy; ancianciulli@unisa.it (A.C.); apacifico@unisa.it (A.P.); savinoquagliarella4@gmail.com (S.Q.); n.bruno5@studenti.unisa.it (N.B.); vaschettino@unisa.it (V.S.); gboccia@unisa.it (G.B.); 2 San Giovanni di Dio e Ruggi d’Aragona University Hospital, 84081 Salerno, Italy; manente392@gmail.com; 3 Integrated Care Department of Health Hygiene and Evaluative Medicine, San Giovanni di Dio e Ruggi d’Aragona University Hospital, 84131 Salerno, Italy; 4 Hospital and Epidemiological Hygiene Unit, San Giovanni di Dio and Ruggi D’Aragona University Hospital, 84131 Salerno, Italy

**Keywords:** artificial intelligence, machine learning, misinformation, infodemic, public health, social media, health communication

## Abstract

**Background/Objectives:** The COVID-19 pandemic highlighted how infodemics—an excessive amount of both accurate and misleading information—undermine health responses. Artificial intelligence (AI) and digital tools have been increasingly applied to monitor, detect, and counter health misinformation online. This scoping review aims to systematically map digital and AI-based interventions, describing their applications, outcomes, ethical and equity implications, and policy frameworks. **Methods:** This review followed the Joanna Briggs Institute methodology and was reported according to PRISMA-ScR. The protocol was preregistered on the Open Science Framework . Searches were conducted in PubMed/MEDLINE, Scopus, Web of Science, and CINAHL (January 2017–March 2025). Two reviewers independently screened titles/abstracts and full texts; disagreements were resolved by a third reviewer. Data extraction included study characteristics, populations, technologies, outcomes, thematic areas, and domains. Quantitative synthesis used descriptive statistics with 95% confidence intervals. **Results:** A total of 63 studies were included, most published between 2020 and 2024. The majority originated from the Americas (41.3%), followed by Europe (15.9%), the Western Pacific (9.5%), and other regions; 22.2% had a global scope. The most frequent thematic areas were monitoring/surveillance (54.0%) and health communication (42.9%), followed by education/training, AI/ML model development, and digital engagement tools. The domains most often addressed were applications (63.5%), responsiveness, policies/strategies, ethical concerns, and equity/accessibility. **Conclusions:** AI and digital tools provide significant contributions in detecting misinformation, strengthening surveillance, and promoting health literacy. However, evidence remains heterogeneous, with geographic imbalances, reliance on proxy outcomes, and limited focus on vulnerable groups. Scaling these interventions requires transparent governance, multilingual datasets, ethical safeguards, and integration into public health infrastructures.

## 1. Introduction

In recent years, public health has been challenged by a phenomenon that spreads faster than viruses themselves: misinformation. In the digital age, the massive overproduction of content, the polarization of debates, and the speed of dissemination across platforms have generated perception distortions and risky behaviors. The World Health Organization (WHO) has defined this scenario as an infodemic, a condition where an overabundance of accurate and false information makes it difficult for citizens to identify trustworthy sources [1]. The COVID-19 pandemic provided the clearest evidence of these dynamics. False claims about unproven treatments, conspiracy theories, and antivaccination campaigns rapidly spread online, undermining trust in institutions and adherence to preventive measures [2,3]. Measurable impacts included reduced vaccination coverage, delayed diagnoses, self-medication with harmful substances, and increased risky behaviors [4,5]. Digital misinformation was further amplified by automated bots, algorithmic recommender systems, and coordinated manipulation strategies, which intensified polarization and eroded confidence in health systems [6,7]. In response, artificial intelligence (AI) technologies have been increasingly applied to counteract misinformation. Natural language processing (NLP) models are used to detect misleading narratives, machine learning and deep learning algorithms classify false content, and chatbots or virtual assistants provide verified health information. In parallel, social listening frameworks enable real-time monitoring of emerging narratives [8,9]. Despite promising results, the literature remains heterogeneous regarding study design, platforms analyzed, outcomes assessed, and methodological quality [10,11]. Beyond technical aspects, ethical and equity concerns remain underexplored. Algorithmic bias, disparities in data access, privacy protection, and transparency issues may exacerbate existing inequalities and contribute to digital divides [12,13]. At the same time, the growing maturity of AI systems—ranging from NLP to deep learning and hybrid human-in-the-loop approaches—highlights both opportunities and risks for integrating digital technologies into public health practice. Given the diversity of approaches and the absence of a unified framework, there is a need for an updated mapping of evidence. This scoping review aims to (i) classify existing studies into five thematic areas—monitoring and surveillance, AI/ML model development, education/training, health communication, and digital engagement—and (ii) examine findings through five cross-cutting domains: applications, responsiveness, ethical concerns, equity/accessibility, and policies/strategic frameworks. To our knowledge, no previous review has comprehensively mapped the integration of AI and digital tools in public health responses to misinformation across multiple domains.

## 2. Materials and Methods

### 2.1. Study Design and Setting

This scoping review was conducted in accordance with the methodological guidance provided by the Joanna Briggs Institute (JBI) for evidence synthesis [14]. Reporting followed the Preferred Reporting Items for Systematic Reviews and Meta-Analyses extension for Scoping Reviews (PRISMA-ScR) checklist [15]. The review protocol was prospectively registered on the Open Science Framework (DOI: 10.17605/OSF.IO/XRTSE). [16]. This review was carried out between February and April 2025 by a multidisciplinary team. Two independent reviewers (S.Q., N.B.) screened and extracted the data, with discrepancies resolved by a senior reviewer (A.C.).

### 2.2. Participants and Eligibility Criteria

Eligible studies included primary research (observational, experimental, qualitative, mixed-methods), reviews, methodological articles, and institutional reports addressing the use of artificial intelligence (AI), machine learning (ML), natural language processing (NLP), or digital platforms (e.g., social media, chatbots, virtual assistants) in the context of health misinformation, infodemics, or digital public health communication. Inclusion criteria required:

1. Population: general population, patients, healthcare workers, or policymakers exposed to health-related misinformation.
2. Concept: application of AI/ML, social media analytics, or digital communication strategies for monitoring, detection, prevention, education, or mitigation of misinformation.
3. Context: public health and health communication at global, regional, or local levels.

Exclusion criteria were editorials without empirical or methodological contribution; non-health-related misinformation studies; and publications without full-text availability in English, Italian, French, or Spanish.

Preprints were not excluded a priori if they met all other eligibility criteria (i.e., primary data, methodological transparency, and relevance to the review objectives).

### 2.3. Information Sources and Search Strategy

Electronic searches were performed in PubMed/MEDLINE, Scopus, Web of Science, and CINAHL. The search covered publications from January 2017 to March 2025. A combination of controlled vocabulary (MeSH terms) and free-text terms was used, including:

(“social media”[MeSH Terms] OR “social media”[Title/Abstract]) AND (“artificial intelligence”[MeSH Terms] OR “AI”[Title/Abstract]) AND (“public health”[MeSH Terms] OR “public health”[Title/Abstract]) AND (ethics OR disinformation OR equity OR “health access”)

The search strategies were adapted for each database, presented in the Supplementary Materials (Table S1). Reference lists of included studies were manually screened to identify additional eligible works (File S1).

### 2.4. Selection Process

All records were imported into Zotero reference manager, and duplicates were removed. Screening was performed in two phases [17]:

1. Title/abstract screening against eligibility criteria.
2. Full-text assessment of potentially eligible studies.

**Figure 1:**
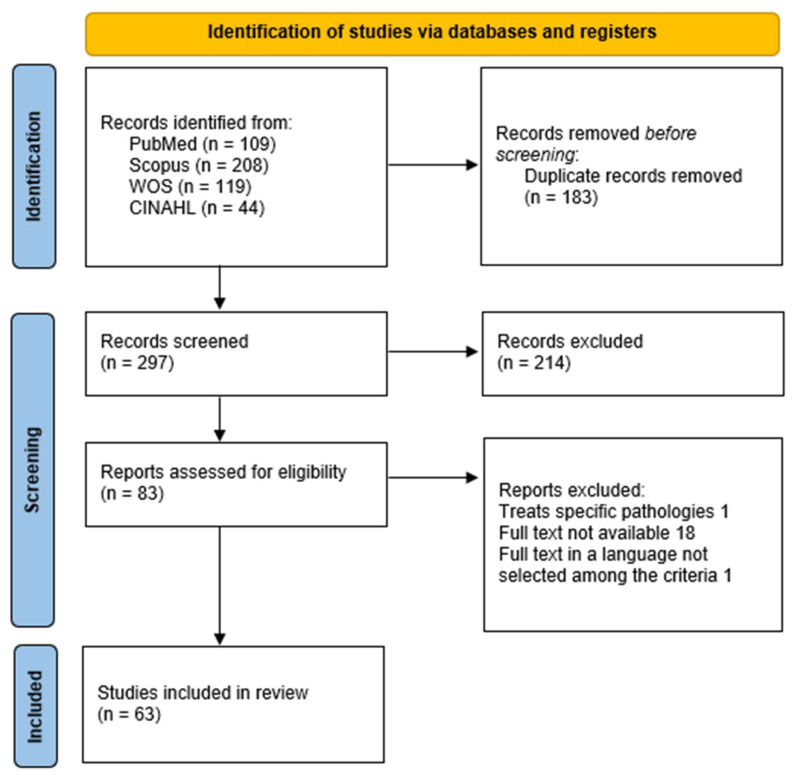
Flow chart of the study selection process.

A PRISMA-ScR flow diagram summarizes the selection process (Figure 1).

Disagreements between reviewers were resolved through discussion and consensus, with arbitration by a senior reviewer (AC) in cases of persistent discrepancies.

Inter-rater agreement was evaluated during the pilot screening phase using Cohen’s kappa coefficient, which yielded a substantial level of agreement (κ = 0.82), confirming the consistency of eligibility judgments prior to full screening.

### 2.5. Data Extraction and Charting

A structured data extraction form was developed and initially piloted on a random sample of five studies to ensure consistency and completeness across reviewers. The final version included 15 predefined variables grouped onto four categories: (i) general study information, (ii) methodological features, (iii) technological and contextual details, and (iv) outcome mapping to thematic areas and transversal domains. Data extraction was conducted independently in duplicate by two reviewers, with discrepancies resolved by consensus and adjudication by a senior reviewer. To prevent duplication across evidence levels, all included records were manually cross-checked during data extraction. When a primary study was also included within a scoping or systematic review, it was counted only once—under the higher evidence category (i.e., review level)—to avoid nested duplicates. Institutional implementation cases were treated as a distinct evidence type and verified separately to ensure mutual exclusivity across categories. Each reviewer populated the standardized extraction sheet in Microsoft Excel, and the compiled dataset was cross-checked for discrepancies through automated cell comparison. The complete codebook, variable definitions, and dataset are available in the Supplementary Materials. Each study could be assigned to more than one thematic area (multi-label classification); therefore, frequencies and percentages reflect tag occurrences rather than mutually exclusive categories. The finalized dataset served as the quantitative basis for the descriptive analyses presented in Tables S2 and S3.

The categorization into five thematic areas and five cross-cutting domains was developed through an iterative inductive–deductive process. The initial structure was informed by recognized digital health and infodemic management models, including the WHO Infodemic Management Framework, the CDC Digital Media Surveillance Model, and the European Commission’s Digital Health Action Plan. These frameworks provided the conceptual foundations for identifying recurring operational and governance dimensions, which were then refined through thematic synthesis of the included studies. The five thematic areas capture the main functional applications of AI and digital technologies, while the five cross-cutting domains reflect systemic, ethical, and policy-oriented dimensions of digital public health transformation.

### 2.6. Outcomes of Interest

The primary outcomes of this scoping review were defined according to five cross-cutting domains that guided data extraction and synthesis:

1. Applications—operational uses of AI and digital tools in infodemic management, including detection, classification, and surveillance of health misinformation.
2. Responsiveness—capacity of interventions to support timely and adaptive public health responses, such as early warning systems, crisis communication, and real-time monitoring.
3. Ethical concerns—issues related to algorithmic bias, transparency, accountability, data protection, and the risk of exacerbating misinformation or inequities.
4. Equity and Accessibility—attention to vulnerable populations, digital divides, multilingual contexts, inclusivity of tools, and accessibility features.
5. Policies and Strategic frameworks—implications for governance, regulatory initiatives, institutional guidelines, and integration of digital tools into public health systems.

Each included study was classified within one or more of these domains, as pre-specified in the codebook.

### 2.7. Data Management and Synthesis

Data were charted into an Excel dataset and analyzed descriptively. Quantitative data were synthesized using frequencies, proportions, and measures of central tendency and dispersion. Sample sizes were reported as median [IQR] and mean ± SD. Proportions were presented with 95% confidence intervals (Wilson method). Given the heterogeneity of designs, a formal meta-analysis was not feasible.

Although formal risk-of-bias assessment is not mandatory in scoping reviews, we conducted a qualitative appraisal of methodological transparency and reporting completeness based on JBI indicators (clarity of objectives, sampling strategy, data source description, and analytical coherence) [14]. This appraisal was used to contextualize the strength of evidence and identify potential gaps without excluding studies from the synthesis.

### 2.8. Statistical Analysis

All data extracted from the included studies were analyzed descriptively. Proportions were reported as n/N (%) with 95% confidence intervals calculated using the Wilson method. Continuous variables (e.g., sample sizes of digital datasets or human participants) were summarized as median [IQR] and mean ± SD, with minimum and maximum values where available. No meta-analysis or quantitative pooling was performed, in accordance with the objectives of a scoping review. Analyses were conducted directly on the standardized extraction table, using established statistical formulas.

## 3. Results

The included studies were published between 2017 and 2025, covering a broad geographical distribution: AMRO 26/63 (41.3%; 95% CI 30.0–53.6), EURO 10/63 (15.9%; 8.9–26.8), WPRO 6/63 (9.5%; 4.4–19.3), EMRO 4/63 (6.3%; 2.5–15.2), SEARO 2/63 (3.2%; 0.9–10.9), and AFRO 1/63 (1.6%; 0.3–8.5); 14/63 (22.2%; 13.7–33.9) had a multinational or global scope. Study designs included 22 observational/infodemiological analyses on social media, 14 model development and validation studies, 9 scoping/systematic reviews, 7 qualitative studies, and 11 institutional implementation cases. Temporal coverage revealed an exponential increase in scientific production starting from 2020, coinciding with the COVID-19 pandemic, which represented the primary driver of research and application of digital tools to counter misinformation.

In studies involving human participants (*n* = 4 with explicitly reported sample size), sample sizes were generally smaller: the median was 150 subjects [IQR 19–362], with a mean of 230 and a standard deviation (SD) of 283. Values ranged from small groups of interviewees (<30 participants, e.g., qualitative designs) to structured surveys with several hundred respondents [18,19].

### 3.1. Main Thematic Areas

The classification of included studies by thematic area is presented in Table S2, showing the predominance of Monitoring and Surveillance (34/63) and Health Communication (27/63).

#### 3.1.1. Monitoring and Surveillance

The thematic distribution revealed a predominance of monitoring and surveillance, with 34 out of 63 studies (54.0%; 95% CI 41.8–65.7) dedicated to social listening, operational taxonomies, and forecasting of information trends [20,21,22,23,24,25,26,27,28,29,30,31,32,33,34,35,36,37,38,39,40,41,42,43,44,45,46,47,48,49,50,51,52]. Research in this area employed digital platforms such as Twitter/X, Facebook, Instagram, YouTube, and Weibo to map the spread of misinformation, analyze its temporal and geographic dynamics, and link them to public health outcomes. Longitudinal analyses demonstrated that peaks in misinformation coincided with crisis events or periods of high media exposure [24,40,43]. Other studies highlighted statistically significant associations between misinformation and health behaviors: in Taiwan, the prevalence of fake news was negatively correlated with weekly vaccination rates [22], while a Weibo-based model accurately predicted the trajectory of severe COVID-19 cases during the Wuhan lockdown [51]. Large-scale institutional projects, such as WHO-EARS, confirmed the scalability of multilingual social listening systems, showing superior performance compared to traditional Boolean filters in terms of precision and recall [41,50]. Additional methodological approaches tested advanced predictive models based on NLP, topic modeling, and network analysis, underscoring their capacity to anticipate clinical demand and identify areas at risk of service saturation [29,32,42]. Overall, this area demonstrates how digital data can serve as real-time epidemiological sensors, complementing traditional surveillance and supporting health preparedness.

#### 3.1.2. AI/ML Model Development

Five studies (7.9%; 95% CI 3.4–17.3) developed and validated predictive models or classifiers based on machine learning and deep learning techniques [19,36,52,53,54]. These works focused on refining algorithmic architectures to detect and classify health-related misinformation content. For example, multimodal models integrating text, images, and metadata achieved recognition of anti-vaccine content on Instagram with an accuracy exceeding 97% [49], while hybrid frameworks such as BERT-LSTM reached accuracy rates of 93.5%. In one study, their implementation was also associated with higher perceived user confidence, although this represents a contextual behavioral outcome rather than a technical performance metric [48]. Other approaches highlighted the importance of incorporating contextual factors, such as informational uncertainty, to enhance model predictability [37], or developed detection pipelines capable of outperforming existing benchmarks [54]. Methodological reviews further synthesized advancements in the field, emphasizing persistent challenges related to imbalanced datasets, lack of transparency, and limited generalizability [36,53]. This body of literature indicates that, despite high technical performance, the transferability of models into operational public health contexts remains a central challenge.

#### 3.1.3. Education and Training

Education and training represented a smaller but significant area, with 6 studies (9.5%; 95% CI 4.4–19.3) [28,55,56,57,58,59]. Evidence shows that targeted educational programs and digital platforms can strengthen users’ critical and digital skills. Academic interventions, such as summer programs in computational research, demonstrated substantial improvements in computing and data analysis competencies among participating students [56], while surveys conducted among university students highlighted how academic training enhances awareness and verification practices for online information [55].

Chatbots and virtual assistants also played an educational and supportive role, fostering greater engagement in identifying and actively countering misinformation [57]. In parallel, conceptual frameworks emphasized the importance of promoting eHealth literacy through AI-based approaches, including automated translation and content simplification [58,59]. Overall, this area illustrates how digital educational strategies can be integrated into public health programs to strengthen informational resilience, though challenges remain regarding sustainability and the heterogeneity of target groups.

#### 3.1.4. Health Communication

Health communication was explored by 27 studies (42.9%; 95% CI 31.3–55.3), which examined digital strategies, AI tools, and institutional communication projects to counter misinformation [27,28,34,39,44,46,47,48,49,60,61,62,63,64,65,66,67,68,69,70,71,72,73,74,75,76,77,78]. Initiatives such as Dear Pandemic demonstrated the ability to reach millions of users through clear language and accessible graphics, increasing trust in scientific communication while countering misleading narratives [60]. Similarly, chatbots and virtual assistants provided validated responses to common public concerns, proving to be low-cost and highly scalable tools [18,63].

Several studies also highlighted how misinformation is closely tied to risk perception and trust in health institutions, underscoring the importance of transparent and culturally adapted communication strategies [65,73]. The use of AI and digital platforms for institutional communication proved particularly valuable during epidemic emergencies and in stigmatized contexts, such as the mpox outbreak [27]. However, the risks of amplifying bias, the sustainability of initiatives, and the need for ethical governance emerged as recurring challenges [44,76,77].

#### 3.1.5. Digital Engagement

Finally, digital engagement was addressed in 4 studies (6.3%; 95% CI 2.5–15.2), which explored the direct involvement of citizens in co-producing content or providing feedback on digital interventions [38,48,79,80]. Interventions targeting adolescents with chronic conditions demonstrated how digital platforms can support therapeutic adherence and mental health, although barriers such as limited access and digital inequality persist [77]. IT surveys and review studies highlighted the potential of participatory technologies in combating misinformation, while stressing the need for open datasets and interdisciplinary approaches [38].

Other contributions underscored the usefulness of digital frameworks to enhance user engagement, for example, through multimodal educational systems [48] or integrated platforms for vaccine communication and distribution [78]. Although this area remains the least developed, it plays a strategic role in building alliances with communities and strengthening collective resilience against misinformation campaigns.

### 3.2. Cross-Cutting Domains

A complementary synthesis by transversal outcome domains is provided in Table S4, with Applications being the most frequent (40/63), followed by Responsiveness (26/63) and Policies/Strategic frameworks (24/63). The mapping of cross-cutting domains showed a clear predominance of applications, addressed in 40/63 studies (63.5%; 95% CI 51.1–74.3). This was followed by responsiveness in 26/63 studies (41.3%; 95% CI 30.0–53.6), policies/strategic frameworks in 24/63 (38.1%; 95% CI 27.0–50.5), ethical concerns in 18/63 (28.6%; 95% CI 18.9–40.7), and equity/accessibility in 14/63 (22.2%; 95% CI 13.7–33.9). As expected in a multi-label scoping review, overlaps were common, with several studies addressing multiple domains simultaneously.

#### 3.2.1. Applications

The applications domain was the most represented, with 40 out of 63 studies (63.5%; 95% CI 51.1–74.3) [20–23; 29–40; 45–49; 50–54; 56,59,60,63,66–68; 72–75; 79]. The studies highlighted a wide range of practical uses of AI and digital tools in public health. For instance, social listening on platforms such as Twitter, Facebook, and YouTube enabled real-time monitoring of public conversations and the identification of misinformation peaks linked to epidemic or media events [20,24,40]. In parallel, predictive models showed the ability to classify misleading content with high accuracy, such as SmartEye [35] or the tri-intelligence framework [52], paving the way for scalable implementation in surveillance systems. Qualitative and conceptual studies also emphasized the role of digital technologies for communication and training: chatbots and virtual assistants improved user engagement and trust [18,64], while initiatives such as Dear Pandemic applied clear and culturally accessible communication strategies with a strong impact on health literacy [60]. Overall, this domain shows that concrete applications are diverse, but their sustainability and replicability depend on data availability and appropriate regulatory frameworks.

#### 3.2.2. Responsiveness

The responsiveness domain was addressed by 26 studies (41.3%; 95% CI 30.0–53.6) [20,22,24,26,27,29,32,33,34,38,41,42,43,45,46,47,50,51,54,57,60,69,71,72,75,76]. Evidence indicates that the use of social media and AI can enhance the timeliness and effectiveness of health responses in emergency contexts. Infodemiological analyses documented how digital signals anticipated variations in vaccination coverage or the incidence of severe clinical cases [22,51]. Institutional social listening projects integrated multilingual taxonomies and interactive dashboards, enabling the identification of information voids and real-time adaptation of communication strategies [41,50]. On the educational side, chatbot-based interventions proved effective in stimulating proactive behaviors against misinformation when designed around situational motivations and gratifications [57]. Collectively, these studies confirm that responsiveness is a distinctive feature of digital solutions, although it remains constrained by data accessibility and the capacity to adapt models to diverse socio-cultural contexts.

#### 3.2.3. Ethical Concerns

Ethical aspects were addressed by 18 studies (28.6%; 95% CI 18.9–40.7), which highlighted critical issues related to privacy, algorithmic bias, and the risk of amplifying misinformation [36,39,43,44,47,58,61,63,66,67,68,69,70,71,72,73,76,78]. Several works emphasized that the lack of transparency in machine learning models can undermine trust and limit institutional adoption [36,44]. Others discussed the risk of stigmatization linked to discriminatory narratives, such as those observed in the context of mpox or in linguistic inequalities [73,74]. Ethical concerns were also raised about the role of platform algorithms in propagating misinformation and the urgent need for shared governance frameworks that balance freedom of expression with the protection of public health [47,66]. Finally, content analysis studies documented the risk of economic exploitation of misinformation, such as the advertising of alternative cancer treatments on Meta platforms [78]. Overall, this domain shows that infodemic management cannot overlook the need for a solid ethical framework and clear accountability mechanisms.

#### 3.2.4. Equity and Accessibility

The equity and accessibility domain included 14 studies (22.2%; 95% CI 13.7–33.9), which examined the impact of digital technologies on vulnerable populations and groups at risk of exclusion [27,55,56,58,59,61,64,68,69,73,74,77,79,80]. Studies conducted among university students and communities with varying levels of digital literacy highlighted that education plays a crucial role in strengthening verification skills and resilience against misinformation [55,56]. Other works underscored how barriers related to language, socio-cultural context, or socioeconomic conditions can exacerbate the effects of the infodemic, requiring culturally adapted and multilingual strategies [61,74]. Interventions targeting adolescents with chronic conditions showed that digital platforms can improve therapeutic adherence, but only when explicitly integrated with attention to equity and digital infrastructure [80]. Overall, this domain emphasizes the need to design inclusive and accessible tools to ensure widespread benefits and reduce inequalities.

#### 3.2.5. Policies/Strategic Frameworks

**Figure 2:**
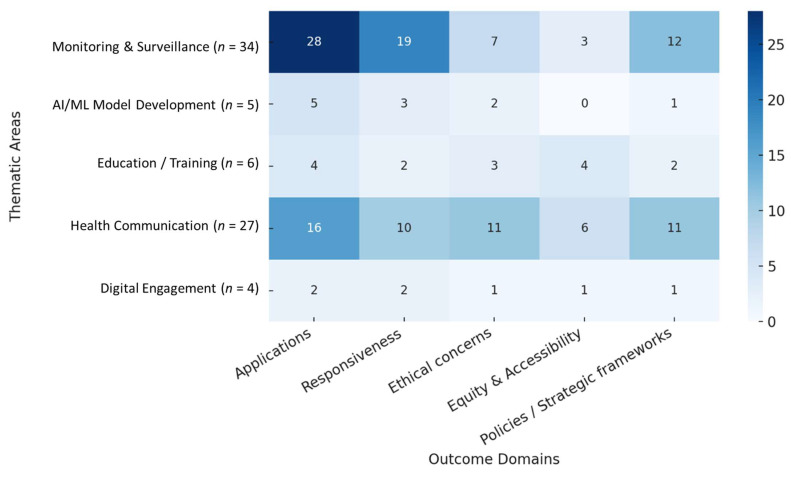
Cross-classification of thematic areas by outcome domains (*n* = 63). Sample sizes and methodological characteristics.

Finally, the domain of policies and strategic frameworks was addressed in 24 studies (38.1%; 95% CI 27.0–50.5) [25,28,38,39,41,44,46,47,48,50,55,58,61,62,65,66,69,70,72,74,75,77,78,79]. These contributions analyzed the role of public policies, regulation, and institutional strategies in tackling the infodemic. Some studies discussed the potential of the Digital Services Act in expanding access to data for research and transparency [77], while others illustrated how platforms such as WHO-EARS can serve as scalable operational infrastructures for global public health [50,75]. Reviews and conceptual studies proposed regulatory and operational frameworks for integrating AI technologies into health systems, emphasizing the need for shared standards and interdisciplinary collaboration [25,28,70]. Recommendations ranged from the adoption of common toolkits for fact-checking [61] to the call for independent audits of machine learning models applied to misinformation [38]. Overall, this domain demonstrates that the effectiveness of digital interventions is closely dependent on the presence of a favorable and coherent regulatory ecosystem. As illustrated in Figure 2, domains were not evenly distributed across thematic areas. Monitoring and Surveillance studies were predominantly linked to Applications and Responsiveness, while Health Communication showed the broadest coverage across domains.

**Figure 3:**
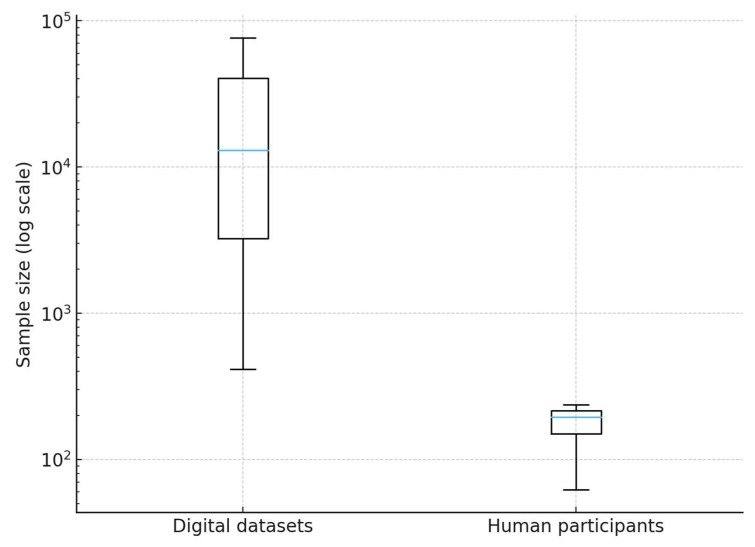
Distribution of sample sizes across included studies.

The studies revealed considerable heterogeneity in dataset sizes (Figure 3).

Across the 40 studies that reported quantifiable digital datasets, the median sample size was 20,875 units [IQR 276–577,294], with a mean ± SD of 10,016,051 ± 36,290,203. This reflects the coexistence of small-scale local analyses and large-scale global studies including over 200 million posts [40,74]. In studies with human participants (*n* = 4 with explicit reporting), sample sizes ranged from small qualitative groups (<30 individuals) to structured surveys with several hundred respondents. The median was 150 [IQR 19–362], with a mean ± SD of 230 ± 283 [55,57].

These findings highlight both the potential and the methodological challenges of working with heterogeneous data sources. While massive datasets allow for timely epidemic signal detection, their reliability depends on transparent governance and rigorous methodological standards. Previous research has emphasized that AI-supported event-based surveillance systems are most effective when built on well-structured and interoperable datasets [42]. Likewise, the very notion of infodemic stresses that information overload—whether accurate or misleading—requires representative and timely datasets to be meaningfully analyzed.

These data confirm a marked asymmetry in sample size distribution, where a limited number of big data analyses generate extremely large values that inflate the mean, whereas the median more accurately reflects the central tendency.

### 3.3. Outcomes

The outcomes reported across the included studies fell into three main categories.

First, algorithmic performance metrics were the most frequently assessed (17 studies), with measures such as accuracy, precision, recall, F1-score, and AUC. Examples include multimodal classifiers for Instagram content achieving accuracies above 97% [49], a BERT-LSTM hybrid model with 93.5% accuracy [48], and the TriIntel framework, which outperformed single approaches in detecting complex misinformation such as sarcasm and conspiracy theories [52].

Second, public health and behavioral indicators were reported in 13 studies, linking digital misinformation signals with real-world outcomes. For instance, the prevalence of fake news showed a negative correlation with weekly vaccination rates in Taiwan [22]; a Hidden Markov Model (HMM) predicted the number of severe and critical COVID-19 cases during the Wuhan lockdown [51]; and several analyses associated misinformation peaks with fluctuations in vaccine intentions [33].

Finally, engagement and reach metrics were described in 6 studies, focusing on dissemination, user interactions, and reach. The “Dear Pandemic” initiative reached over four million monthly views, significantly enhancing scientific literacy [60], while the CoronaAI chatbot documented that 21% of its interactions addressed misinformation-related queries [18].

Together, these outcomes illustrate both the technical feasibility of AI-based tools and their potential to influence population-level health behaviors and literacy.

### 3.4. Synthesis of Findings

Overall, the mapping of included studies shows a rapidly growing use of AI and digital tools to counter health-related misinformation, with applications ranging from real-time surveillance to trust-building initiatives, from healthcare professional training to predictive modeling. At the same time, recurring challenges emerge, including dataset heterogeneity, algorithmic bias, risks of digital exclusion, and the absence of shared international governance frameworks.

The complete list of the 63 studies included in this review, with full bibliographic details and DOIs, is available in Supplementary File List of Included Studies.

## 4. Discussion

This scoping review achieved its aim of systematically mapping the use of AI and digital tools in addressing the infodemic, providing an updated and quantitatively robust overview of the available evidence.

Unlike previous reviews that primarily focused on social media communication dynamics [47] or policy-level recommendations to mitigate infodemics [28], this scoping review offers a broader and more integrated perspective on how AI and digital technologies have been employed in public health responses between 2017 and 2025. Some studies have limited their scope to early COVID-19-related social media data [28,47], examining institutional strategies without quantifying technological applications [28], our study systematically maps the interaction between AI/machine learning models, digital communication tools, and infodemic management across multiple domains.

Furthermore, recent engineering-oriented studies [81,82,83] have focused on the performance of AI models or IoT-based diagnostic applications for COVID-19, but have not addressed their integration into public health governance or misinformation management.

In contrast, several studies identified in our review [20,44,58] illustrate the emerging intersections between AI-based surveillance, digital health literacy, and governance frameworks, highlighting the evolution from technical sensing towards holistic public health system responses. This work, therefore, connects computational and behavioral evidence through a dual analytical framework of five thematic areas and five cross-cutting domains, offering a multidimensional understanding of how digital technologies contribute to infodemic control and health system resilience. The five-by-five analytical framework (Figure 2), proposed in this review advances existing conceptual models by integrating both operational and governance dimensions into a unified analytical structure.

Previous frameworks, such as the WHO Infodemic Management model and the European Digital Health Strategy, have typically addressed communication, surveillance, and ethics as separate pillars [84]. In contrast, our framework systematically connects these elements, enabling multidimensional mapping of AI and digital health applications that encompass performance, responsiveness, equity, and policy coherence.

This integrative structure therefore extends earlier conceptualizations by offering a structured yet flexible taxonomy that can support future evidence syntheses and guide digital health policymaking toward more ethical, equitable, and transparent implementation of artificial intelligence in public health.

The main findings highlighted five key points: the centrality of monitoring and surveillance, which accounted for about half of the included studies; the progressive maturation of AI models, with high performance but persistent issues of generalizability; the potential of educational and communication strategies, including chatbots, to strengthen digital and health literacy; the crucial relevance of equity and ethics as structural rather than accessory dimensions; and the emergence of international regulatory frameworks that are redefining data access and digital governance.

These findings directly respond to the stated objectives of this review, providing both quantitative (with 95% CI) and narrative insights into thematic areas and cross-cutting domains. The role of social media as “digital sensors” for public health is supported by recent studies documenting how temporal and geographic analyses of online conversations can anticipate variations in vaccination coverage and healthcare demand [49,50,74,75,76,79]. Likewise, predictive models based on informational signals have proven useful in detecting waves of misinformation or peaks of clinical cases [51].

This review confirms the high accuracy of multimodal and hybrid models reported in the literature [49,54], while also showing that limited linguistic and geographical datasets reduce replicability. In addition, frequent policy and algorithmic changes introduced by major social media platforms (e.g., Twitter/X, Facebook, YouTube) between 2017 and 2025 have significantly affected data accessibility and comparability. The progressive restriction of APIs, evolving moderation practices, and removal of legacy datasets have introduced temporal variability in data completeness and, consequently, in the quality and reproducibility of infodemiological analyses. This is consistent with studies stressing the need for multilingual and culturally diverse data sources [74,79]. On the communication front, initiatives such as the “Dear Pandemic” project [60] have demonstrated how clear language, visuals, and translations can enhance reach and trust, consistent with research on evidence-based communication strategies [5]. Educational initiatives have also been shown to improve computational and fact-checking skills, though at a significant organizational cost [56].

Finally, the strengthening of regulatory frameworks—such as the European Digital Services Act [77] and the global WHO-EARS system [50]—represents an evolution consistent with recent policy analyses [44].

Three overarching mechanisms emerge from this review:

1. Informational epidemiology: digital signals can anticipate epidemic trends and behaviors, serving as proxies for public health preparedness.
2. Technology and literacy: AI models, when integrated with educational and communication strategies, can enhance health literacy and resilience against misinformation.
3. Governance and trust: the legitimacy and adoption of digital tools depend on transparency, independent audits, and the inclusion of vulnerable groups.

These dynamics have important practical implications. Public health services can incorporate social listening dashboards based on standardized taxonomies to identify emerging narratives in real time; complement multimodal classifiers with human oversight for complex content (e.g., memes, sarcasm, images); activate clinically governed chatbots to address recurring informational needs; and develop culturally adapted digital literacy programs targeting vulnerable groups.

For research, comparative testing of multimodal models in multilingual and multicultural contexts is needed, along with the development of ethical governance frameworks and independent auditing to ensure transparency and accountability. Future studies should also explore the real-world impact of these technologies on health outcomes (e.g., vaccination coverage, treatment adherence, reduction in stigma) and should include greater involvement of healthcare professionals, including nurses, in the design and implementation of digital tools.

From a policy perspective, this review provides several concrete insights for decision-makers. Governments and public health agencies can leverage AI-driven social listening platforms (such as WHO-EARS or machine learning-based dashboards) to detect and respond to emerging misinformation trends in real time. Natural language processing (NLP) classifiers and predictive AI models can support early identification of false narratives and anticipate peaks in misinformation or shifts in public sentiment, improving crisis preparedness and communication planning. Chatbots and virtual assistants, when clinically validated and ethically governed, can serve as scalable and low-cost communication tools for public engagement, particularly during vaccination campaigns or chronic disease programs. These tools, when embedded within transparent and equitable regulatory frameworks, can help policymakers strengthen digital health resilience while safeguarding privacy, inclusivity, and trust.

**Strengths and limitations**

This scoping review presents several strengths. First, it was conducted following PRISMA-ScR and JBI guidance, with protocol preregistration on OSF, ensuring methodological transparency and reproducibility. Second, the dual classification by thematic areas and outcome domains allowed for structured and quantitative mapping, reinforced by 95% confidence intervals. Third, the integration of both descriptive statistics and narrative synthesis provided a comprehensive overview of digital and AI-driven responses to health misinformation.

However, some limitations should be acknowledged. The heterogeneity of study designs and outcomes limited the comparability of findings, and most studies reported proxy indicators (e.g., accuracy, engagement) rather than direct clinical outcomes. The evidence was geographically imbalanced, with an overrepresentation of studies from AMRO and limited contributions from AFRO and SEARO regions. Moreover, language restrictions and reliance on peer-reviewed literature may have led to exclusion of relevant gray literature. In addition, the inclusion of the keyword “public health” in the search strategy may have inadvertently excluded relevant AI-detection or modeling studies published in engineering or computer science journals that do not explicitly identify as public health–related. This potential selection bias is acknowledged as a methodological limitation. Finally, no formal risk-of-bias assessment was performed, consistent with scoping review methodology, but this precludes judgments on the quality of individual studies.

The geographical imbalance identified in this review deserves further consideration. Most of the included studies originated from high-income regions—particularly the Americas (AMRO) and Europe (EURO)—while evidence from Africa (AFRO) and South-East Asia (SEARO) was markedly limited. This underrepresentation reflects structural disparities in digital infrastructure, data accessibility, and research capacity across regions.

As a result, the generalizability of the findings is constrained: AI-based and digital interventions validated in data-rich settings may not be directly transferable to low- and middle-income countries, where linguistic diversity, limited connectivity, and differing patterns of health misinformation can affect model performance and adoption.

Addressing this imbalance requires strengthening international collaborations, promoting multilingual and culturally adapted datasets, and fostering equitable participation of underrepresented regions in digital public health research.

## 5. Conclusions

This scoping review demonstrates that artificial intelligence and digital tools are no longer futuristic solutions, but already operational technologies that can strengthen information surveillance, public communication, and health literacy. The integration of social listening dashboards, multimodal predictive models, and educational chatbots can contribute to a more timely and effective response to infodemic crises.

However, the available evidence remains heterogeneous, with marked geographical imbalances and limited attention to vulnerable populations. Scalability and widespread adoption require algorithmic transparency, equity in access, data interoperability, and training programs for healthcare professionals.

The findings provide practical guidance for policymakers, health institutions, and the scientific community, underscoring the urgency of developing ethical and sustainable digital strategies that enhance the resilience of public health systems against misinformation. This review highlights that future preparedness requires not only technological innovation but also equitable governance to prevent infodemics from undermining global health security.

## Data Availability

De-identified data are openly available in the Open Science Framework (OSF) at https://doi.org/10.17605/OSF.IO/XRTSE, (accessed on 15 October 2025). Additional materials are available from the corresponding author upon reasonable request.

## Supplementary Materials

The following supporting information can be downloaded at: https://www.mdpi.com/article/10.3390/healthcare13202623/s1, Table S1: Search strategy; Table S2: Data extraction; Table S3: Distribution of thematic areas across the 63 included studies; Table S4: Distribution of outcome domains across the 63 included studies; File S1. List_of_Included_Studies.
