# Supplementary material for: Artificial Intelligence and Digital Technologies Against Health Misinformation: A Scoping Review of Public Health Responses
Source: Healthcare (Basel). 2025 Oct 18;13(20):2623. doi: 10.3390/healthcare13202623 (PMC12564032; doi:10.3390/healthcare13202623)

Table S1. Search strategy

1) PubMed/MEDLINE

Ricerca	Azioni	Dettagli	Domanda	Risultati	Tempo
#3	•••	>	Cerca: **(("social media" O "siti di social network" O "piattaforme social" O "comunità online") E ("intelligenza artificiale" O AI O "apprendimento automatico" O "apprendimento profondo" O "elaborazione del linguaggio naturale") E ("salute pubblica" O "salute della popolazione" O "promozione della salute" O "comunicazione sanitaria" O "monitoraggio epidemiologico" O "risposta alle epidemie") E ("disinformazione" O "disinformazione" O "infodemia" O "accuratezza delle informazioni sanitarie" O "alfabetizzazione sanitaria")) E ("2018"[Data - Pubblicazione] : "2025"[Data - Pubblicazione])** Filtri: **Umani**	109	09:16:35

2) Scopus

Ricerca avanzata

< Ricerca di base Avanzato

Suggerimenti per la ricerca ?

Inserisci la stringa di query

TITLE-ABS-KEY(("social media" O "siti di social network" O "piattaforme social" O "comunità online") E ("intelligenza artificiale" O AI O "apprendimento automatico" O "apprendimento profondo" O "elaborazione del linguaggio naturale") E ("salute pubblica" O "salute della popolazione" O "promozione della salute" O "comunicazione sanitaria" O "monitoraggio epidemiologico" O "risposta alle epidemie") E ("disinformazione" O "disinformazione" O "infodemia" O "accuratezza delle informazioni sanitarie" O "alfabetizzazione sanitaria")) E PUBYEAR > 2017 E PUBYEAR < 2026

Query di riepilogo | Aggiungi il nome dell'autore / Affiliazione | Forma chiara | Ricerca

ALL("Architetture cognitive") E NOME-AUTORE(smith)

TITOLO-ABS-CHIAVE(*reclamo somatico donna) E PUBYEAR DOPO 1993

SRCTITLE(*campo ornith*) E VOLUME(75) E NUMERO(1) E PAGINE(53-66)

Tipo	Query di ricerca e risultati	Banca dati	Risultati	Azioni
Sessione corrente	Esportare			
Ricerca	TS=("social media" O "siti di social network" O "piattaforme social" O "comunità online") E TS=("intelligenza artificiale" O AI O "apprendimento automatico" O "apprendimento profondo" O "elaborazione del linguaggio naturale") E TS=("salute pubblica" O "salute della popolazione" O "promozione della salute" O "comunicazione sanitaria" O "monitoraggio epidemiologico" O "risposta alle epidemie") E TS=("disinformazione" O "disinformazione" O "infodemia" O "accuratezza delle<br>17:20	Raccolta di base di Web of Science Mostra edizioni	119	

3) Web of Science

Available from: https://wwsummary/f83fe04e-de79-43b1-9fb6-a5fdde7f0352-01472466fe/relevance/1w.webofscience.com/wos/woscc/

CINAHL

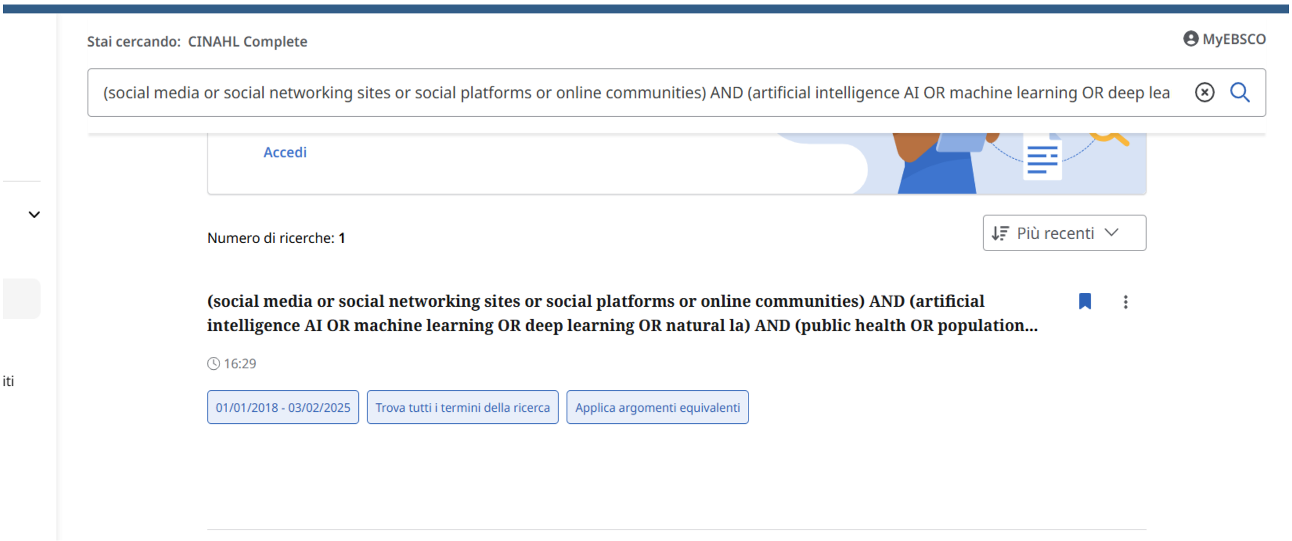

Supplement: Supplementary file 1 [file healthcare-13-02623-s001.zip › 01. Supplementary Search strategy (Table S1).pdf]
