# Supplementary material for: Artificial Intelligence and Digital Technologies Against Health Misinformation: A Scoping Review of Public Health Responses
Source: Healthcare (Basel). 2025 Oct 18;13(20):2623. doi: 10.3390/healthcare13202623 (PMC12564032; doi:10.3390/healthcare13202623)
Supplement: Supplementary file 1 [file healthcare-13-02623-s001.zip › 03. Supplementary Tables S3 and S4.pdf]

Table S3: Distribution of thematic areas across the 63 included studies

Thematic Area	Quantitative signal (n/N, % [95% CI])	Main finding	Representative examples	Practical implication
Monitoring & Surveillance	34/63, **54.0%** [41.7–65.9]	Social media and AI used as **real-time sensors**: infodemic peaks tracked alongside crises, predictive models linked signals to vaccination rates or severe cases.	Boatman, 2024; Chen YP, 2022; Yan, 2022; White BK, 2023; Pobiruchin, 2020; Rees, 2019	Integration of **social listening dashboards** with PH surveillance and early-warning systems.
AI/ML Model Development	5/63, **7.9%** [3.4–17.3]	Focused on building/validating models (multimodal, BERT-LSTM, uncertainty frameworks) with high accuracy (>90%).	Di Sotto, 2022; Sharifpoor, 2025; Zhang, 2024; Wang Z, 2021	Promising tools for **automated classification**; need external validation and diverse datasets.
Education / Training	6/63, **9.5%** [4.4–19.3]	Interventions with students, professionals, or public to strengthen **digital/health literacy** and fact-checking skills.	Antoliš, 2024; Brown, 2022; Cheng, 2025; Olusanya, 2021	Training programs (academic & community) can reduce susceptibility but require resources and sustainability.

Health Communication	27/63, **42.9%** [31.0–55.6]	Emphasis on **messaging strategies**, chatbot adoption, institutional comms, and campaigns against misinformation.	Albrecht, 2022; Bin Naeem, 2021; Fabrizzio, 2023; Garett, 2021; Tsao, 2021; Tuccori, 2020; Zenone, 2023	Co-design **clear, accessible, and culturally tailored communication**; regulate misinformation in ads.
Digital Engagement	4/63, **6.3%** [2.5–15.2]	Participation-focused studies: adolescents, communities, multilingual/mHealth platforms, and civic engagement.	Crowley, 2024; Zang, 2023; Wang J, 2025	Explore **interactive and participatory approaches** to strengthen trust and co-production.

Table S4: Distribution of outcome domains across the 63 included studies

Outcome / Domain	Quantitative signal (n/N, % [95% CI])	Main finding (what the evidence shows)	Representative examples	Practical implication	Gaps / limitations
Applications	40/63, **63.5%** [51.1–74.3]	Predominance of **operational uses** of AI/social media: automated detection/classification (accuracy often 0.80–0.97), content curation, dashboards, and chatbot use in PH communication.	Abonizio, 2023; Du, 2021; Guo, 2024; Ismail, 2023; Liu J, 2022; Wang Z, 2021; White BK, 2023; Ghenai, 2017; Chen S, 2021; Pobiruchin, 2020	Readiness to **embed ML/NLP pipelines and chatbots** in routine PH workflows for monitoring and outreach.	**Generalizability** limited by platform/language; scarcity of prospective impact evaluations on real PH outcomes.
Responsiveness	26/63, **41.3%** [30.0–53.6]	Social listening + analytics **anticipate or track crises** (misinformation spikes linked to events; signals correlate with vaccination uptake or severe cases).	Boatman, 2024; Edinger, 2023; Chen YP, 2022; Yan, 2022; Purnat, 2021; Rees, 2019; Lanyi, 2022; Temiz, 2023	Integrate **alert thresholds** and event-based playbooks; use dashboards to trigger **timely risk comms** and resource allocation.	Need **standard operating procedures**, validation against gold-standard surveillance, and clarity on **response SLAs**.
Ethical concerns	18/63, **28.6%** [18.9–40.7]	Recurrent issues: **bias/fairness**, privacy/data governance, explainability; risk of **stigma** amplification and unintended harms.	Röchert, 2021; Malecki, 2021; Tasnim, 2020; Patel, 2023; Sallam, 2023; Zhang, 2024; Rodrigues, 2024	Require **transparent model audits**, DPIAs, human-in-the-loop review for high-risk decisions, and harm-mitigation protocols.	Few **formal audits**, limited reporting on **model explainability**, and rare **equity impact assessments**.
Equity & Accessibility	14/63, **22.2%** [13.7–33.9]	Equity appears **under-addressed**: linguistic/cultural tailoring and accessibility features are uneven; vulnerable groups face digital gaps.	Valdez, 2023; Wang Y, 2024; Crowley, 2024; Fabrizzio, 2023; Antoliš, 2024; Wehrli, 2024; Olusanya, 2021; Brown, 2022	Co-design with **priority populations**; **multilingual** taxonomies; accessibility (e.g., low-bandwidth, ASL, plain language).	Sparse **stratified analyses** by sociodemographics; limited **LMIC** representation; few **effectiveness** studies in vulnerable groups.

Policies / Strategic frameworks	24/63, **38.1%** [27.1–50.4]	Growth of **frameworks/regulation**: WHO-EARS methods, data-access debates (DSA), institutional strategies for infodemic management.	White B, 2023; White BK, 2023; Gentili, 2023; Wehrli, 2024; Rodrigues, 2024; Thomas, 2021; Papanikou, 2025; Fernandez-Luque, 2018	Align programs to **standardized taxonomies**, clarify **data-access & accountability**, and plan **capability building**.	Implementation guidance still **heterogeneous**; **data-sharing** and cross-platform interoperability remain challenging.

Methodological note. Proportions and 95% CIs (Wilson) calculated on N = 63 studies: Applications 63.5% [51.1–74.3]; Responsiveness 41.3% [30.0–53.6]; Ethical concerns 28.6% [18.9–40.7]; Equity & Accessibility 22.2% [13.7–33.9]; Policies 38.1% [27.1–50.4].
